# Association of Picky Eating with Growth, Nutritional Status, Development, Physical Activity, and Health in Preschool Children

**DOI:** 10.3389/fped.2018.00022

**Published:** 2018-02-12

**Authors:** Hsun-Chin Chao

**Affiliations:** ^1^Division of Gastroenterology, Department of Pediatrics, Chang Gung Children’s Hospital, Chang Gung Memorial Hospital, Chang Gung University College of Medicine, Taoyuan, Taiwan

**Keywords:** picky eating, growth, development, physical activity, health, preschool children

## Abstract

**Background:**

This study aimed to assess the prevalence of picky eating among preschool children and to evaluate the association between eating behavior and growth, physical activity, development, and health status.

**Methods:**

A structured questionnaire was used to conduct a cross-sectional descriptive study of 300 primary caregivers of children aged 2–4 years in Taiwan. Data collected included: demographics, food preferences, eating behavior, body weight, and height, development, physical activity, and records of medical illness. Data from children defined as picky or non-picky eaters based on parental’ questionnaire responses were analyzed and compared using standard statistical tests.

**Results:**

The mean age of the children was 2.95 years; 162 (54%) were picky eaters. Compared with non-picky eaters, *z*-score of weight-for-age, height-for-age, and body mass index (BMI)-for-age in picky eaters was 0.91, 0.73, and 0.44 SD lower, respectively. There were significant differences of rates in the weight-for-age, height-for-age, and BMI-for-age percentiles <15, between picky and non-picky eaters (*P* = 0.04, 0.023, and 0.005, respectively). Fear of unfamiliar places, poor physical activity, constipation, and high frequency (>2 times in the past 3 months) of medical illness were significantly higher in picky eaters (*P* = 0.01, 0.001, 0.044, and <0.001, respectively).

**Conclusion:**

The prevalence of picky eaters in preschool children was high, resulting in significant detrimental impacts on growth, nutritional status, development, physical activity, and health status.

## Introduction

Picky eating in children a collective term that usually denotes having strong food preferences, consuming an inadequate variety of foods, restricting the intake of some food groups, eating a limited amount of food, or being unwilling to try new foods. Picky-eating behaviors are common in infancy and childhood (1); however, there is no specific medical definition for the term “picky eater” (2–4).

In one study, 20–60% of young children were reported by their parents not to be eating optimally (5). Another study of 120 children aged 2–11 years identified 39% as picky eaters (6), and picky eating prevalence as high as 50% was reported in children aged 19–24 months in a study carried out in North America (7). Picky eating behavior was reported by parents in half (54%) of the Chinese preschoolers aged 3–7 years (8). A recent study reported that 36% of young Chinese preschoolers (24–35-month-old) had picky-eating behaviors (9).

Picky eating can occur in normally developing children as well as in those with medical or developmental disorders (5), and eating disorders in adolescence and early adulthood can sometimes trace back to picky eating in early childhood (10). Behavioral feeding disorders may be associated with suboptimal development (8, 11, 12), and some children who refuse food or are picky have poor weight gain (3, 8, 12).

Picky eating habits of preschool children are understudied worldwide. To date, there has been limited research on picky eating in preschoolers. Therefore, this study set out to determine the prevalence of picky eating among preschool children and to evaluate the association between picky eating and growth, nutritional status, development, physical activity, and health status.

## Materials and Methods

### Participants

This cross-sectional descriptive study used a structured questionnaire to elicit information from parents in Taiwan who were primary caregivers to children aged 2–4 years. Participants were randomly selected from nursery schools and kindergartens in three major cities [Taipei (northern city), Taichung (middle city), and Kaohsiung (southern city)] to meet pre-specified quotas for race, age, and sex, representative of the national population. A total of 300 participants (100 from each city) who met the eligibility criteria were enrolled.

#### Interviewers

The interviewers employed were those who had good education level (college or higher), experience of interviewers on similar studies qualified interviewing skills including language skills, conversation skills (e.g., speech level, comprehension level), organizational skills, and reading skills (e.g., reading loud), and ability to handle potentially emotional or stressful interactions with respondents.

The qualified interviewers were provided general interviewer training for 8 h. The training included standard question-asking, conventions, and clarification of the questionnaires, and unbiased technical skills of being neutral to the respondents, avoidance of using their own definitions to any phrase, or question in the questionnaire, attempt of encouragement in answers that were more complete, and thoughtful, and providing their respondents with appropriate feedback. The interviewers were asked to keep confidential in all identifying information of respondents, as well as respondents’ answers to survey questions.

#### Interview Process

All the data collected from the study was obtained through face-to-face interviews with the children’s caregivers. The participants received the initial screen for their eligibility. The study excluded children with chronic illnesses affecting eating habits and growth status, such as: prematurity, low birth weight (<2,500 g), dental diseases, organic diseases, mental disorders, genetic diseases, psychiatric illness, anorexia, gastro-esophageal reflux disease, esophagitis, food allergies, and lactose intolerance. Families were also excluded if they had limited economic means to support their children’s diets, had inadequate concept for children’s nutritional support, development, and physical activities, or were unable to provide adequate nutrition for other reasons.

All participants were interviewed to gather socio-demographic data, as well as weight and height for age. The interview process incorporated assessment of food preferences, eating behaviors, general development, physical activity, and health questionnaire, and health status by questionnaires. The interviews took approximately 20–30 min to complete. Socio-demographic data were collected using a parental form, which included questions about educational level and family size (number of children). The food, general health, and medical questionnaires mostly comprised closed-ended categorical questions, with listed options for respondents to select. The main questions included children’s food preferences, dietary habits, parent/child interactions during meal-times, language ability, developmental behaviors, level of physical activities, and records of medical illness in the past year. As the survey was quantitative, no detailed discussions were conducted. Trained interviewers carried out the interviews between August 1 and September 31, 2008. All parents or guardians of children who were participants of the investigation provided their written informed consent.

### Assessment of Growth and Nutritional Status

Children’s weight and height were measured outside the classroom on an individual and solicited basis, before the interview associated with the food frequency questionnaire. Children were weighed without shoes and wore light clothing. These measurements were used to derive body mass index (BMI) [weight (kg)/height (m^2^)]. To evaluate the impact of picky eating on growth and nutritional status, the percentile and *z*-score of weight-for-age, height-for-age, and BMI-for-age were assessed.

Weight-for-age, weight-for-height, and height-for-age were expressed as sex- and age-specific percentiles, and the growth standards for height, weight, and BMI based on a general Taiwanese population were used to obtain *z*-scores for each measurement according to age and sex (13).

Parameters used to assess growth status were height-for-age and weight-for-age *z*-scores, and those used to evaluate nutritional status were weight-for-age and BMI-for-age *z*-scores. Weight-for-age, height-for-age, and BMI-for-age percentiles <15 were deemed to indicate that the child was underweight, of short stature, or suffering from malnutrition, respectively.

### Assessment of Picky Eating

The questionnaire for food preferences and eating behaviors addressed two general areas (food preferences and eating behaviors).

#### Food Preferences

In a structured questionnaire, all parents were asked about their child’s food preferences. Questions about preferences for food and food types included a modified version of a questionnaire based on the United Kingdom Department of Health Survey of the Diets of British School Children (14) and dietary assessment among school-aged children (15). The modification of the questionnaire was based on Taiwanese dietary culture and food habits. The questionnaire included two major items: (1) child’s foods (meals) and their preferences in seven food categories: (i) grains (rice, bread, cereals, potato, noodles, etc.), (ii) protein foods (meats, fish, seafood, beans, etc.) (iii) vegetables, (iv) fruits, (v) dairy foods (milk, cheese, yogurt, etc.), (vi) fats and oils (vegetable oil, butter, cream, salad, etc.), and (vii) snacks and sweets (candy, cookie, cake, etc.) in past 2 weeks, (2) preferences in familiar foods (list of 40 foods for regular meals). The answers were “tried and not tried” in each food and responding to preferences of the tried foods. Items were scored on a five-point scale as “like very much,” “like moderately,” “neither like nor dislike,” “dislike moderately,” and “dislike very much.”

#### Feeding and Eating Behaviors

A separate section asked parents about their feeding behaviors (six items: four appropriate behaviors, two inappropriate behaviors) and their child’s eating behaviors (six items: two healthy eating behaviors, four picky eating behaviors). The eating behavior questionnaires were inspired from the Children’s Eating Behavior Questionnaire developed by Wardle et al. (16), the classification of feeding disorders of infancy and early childhood by Chatoor and Ammaniti (10) and a study about the trends of eating behaviors in preschool children (17). The four questions for picky eating behaviors included (i) eats limited foods (usually eat fixed foods or have strong like with regard to food, such as cooked foods, milk, or sweets), (ii) unwillingness to eat regular meals, (iii) unwillingness to try new foods, and (iv) refusal of one or multiple food groups in six major food groups (grains, protein foods, vegetables, fruits, dairy foods, and fats and oil). Items were scored on a five-point scale as “never,” “rarely,” “sometimes,” “often,” or “always.” Mean scores were calculated for each subscale (range 1–5) with higher scores indicating higher values of each trait.

Picky eating was defined as a positive response of “always” to at least one item of the picky eating behaviors on questionnaire of eating behaviors.

### Assessment of Development

The questionnaire for assessment of development was modified based on the Denver Developmental Screening Test II to screen children’s development in four areas of functioning: fine motor-adaptive, gross motor, personal-social, and language skills. Statements relating to development requested respondents to rate their degree of agreement on a five-point scale (unacceptable, improvement expected, acceptable, exceeding expected, outstanding). Mean scores were calculated for each subscale (range 1–5) with higher scores indicating higher values of each trait.

Development was evaluated based on the outcomes of three categories (eight items): learning ability (two items: attention and learning); verbal development (three items: verbal development, language learning, confluence in speech); and interpersonal relationships (three items: adaptation to new environments, cooperation, adaptation of being separated from relatives). The answers of “unacceptable” or “improvement expected” of the item assessed were considered as having slow, poor, or deficient development. Those participants with one or more of low quality items in each category of development were defined as having low quality general development (learning disability, poor verbal development, or negative interpersonal relationships).

### Assessment of Physical Activity

The questionnaire for assessment of physical activity was modified based on a study of objective measurement of physical activity and sedentary behavior (18). Statements relating to physical activity requested respondents to rate their degree of agreement on a five-point scale (unacceptable, improvement expected, acceptable, exceeding expected, outstanding). Mean scores were calculated for each subscale (range 1–5) with higher scores indicating higher values of each trait. The questionnaire assessing physical activity consisted of four items: normal-pace walking; sport activities; stair-climbing; and running. The answers of “unacceptable” or “improvement expected” of the item assessed were considered as having low level physical activity. Those with two or more low-level physical activities were defined as having a poor general physical activity level.

### Assessment of Health Status

The health status was assessed by the records of medical illness. The medical illness evaluation included constipation and acute infectious illnesses (as well as the frequency of such illnesses, particularly within the past 3 months).

### Statistical Analyses

Data entry and data processing were carried out using SAS statistical software (SAS Inc., Chicago, IL, USA) for behavioral items on the questionnaire. Both descriptive and inferential data analyses were applied using appropriate statistical tests of significance (Chi-square, Student’s *t*-test). Numerical data were analyzed using the Student’s *t*-test, and categorical data were analyzed using the Chi-square analysis. A confidence interval (CI) of 95% and a significance level of *P*-value <0.05 were used.

### Ethics Approval

Institutional Review Board of the Human Research Committee of Chang Gung Memorial Hospital approved the study protocol (Reference 97-0858B).

## Results

### Demographic Data and Characteristics

Three hundred and fifty-eight participants (Taipei city: 121; Taichung city: 119; Kaohsiung: 118) were screened, of whom 300 met eligibility criteria were enrolled. Of the 58 excluded participants, 31 children had chronic illnesses affecting eating habits and growth status, 15 caregivers had limited economic means to support their children’s diets, and 27 caregivers did not have enough concept for children’s nutrition support, development, and physical activities or were unable to provide adequate nutrition for other reasons.

Table 1 summarizes the demographic differences between picky and non-picky eaters. 300 were enrolled. Based on the food and dietary questionnaire survey, 162 children (54%) were found to have picky-eating behavior. The mean age of these children was 2.97 ± 0.59 years. There were no statistical differences in sex, age, primary caregiver, education levels of caregiver, or family size between picky and non-picky eaters.

**Table 1:** Demographic characteristics of picky versus non-picky eaters.

Variables:*n*(%)	Picky (*n* = 162)	Non-picky (*n* = 138)	95% CI^a^	*P*
			χ^2b^	
**Children**				
Sex				
Male: 156 (52)	83 (51.2)	73 (52.9)	0.029^b^	0.864
Female: 144 (48)	79 (48.8)	65 (47.1)		
Age (years): mean (SD)	2.97 (0.59)	2.93 (0.61)	−0.866, 0.166^a^	0.533
2–3: 159 (53)	87 (53.7)	72 (52.2)	0.022^b^	0.882
3–4: 141 (47)	75 (46.3)	66 (47.8)		

**Caregiver**				
Father: 36 (12)	21 (13)	15 (10.9)	0.143^b^	0.706
Mother: 264 (88)	141 (87)	123 (89.1)		

**Education**				
High school: 93 (31)	49 (30.3)	44 (31.9)	0.033^b^	0.857
College: 171 (57)	92 (56.8)	79 (57.2)	0.001^b^	0.970
Masters or PhD: 36 (12)	21 (12.9)	15 (10.9)	0.143^b^	0.706

**Family size**				
One child: 193 (64.3)	109 (67.3)	84 (60.9)	1.071^b^	0.301
>One child: 107 (35.7)	53 (32.7)	54 (39.1)		

*^a^Student’s t-test*.

*^b^Chi-square (χ^2^) analysis*.

*CI, confidence interval*.

The most common typical behaviors of a picky eater among the group responding “always” included: being unwilling to eat regular meals (18.5%); refusing food, particularly fruit and vegetables (16.7%); eating sweets or snacks instead of meals (14.8%); being unwilling to try new foods (14.2%); excessive drinking of milk (14.2%), and accepting only a few types of food (13.6%). The picky eaters did not like to eat meat (59 cases, 37.1%), vegetables (63 cases, 38.9%), fruit (36 cases, 22.2%), and specific kinds of vegetables or fruit (35 cases, 21.6%). Compared to non-picky group, significant lower number of accepted foods and lower score in food preference were found in picky group (15.1 ± 3.7 vs. 26.7 ± 4.1, *P* < 0.001; 3.0 ± 1.3 vs. 3.6 ± 1.1, *P* < 0.001).

### Anthropometric Data

**Figure 1:**
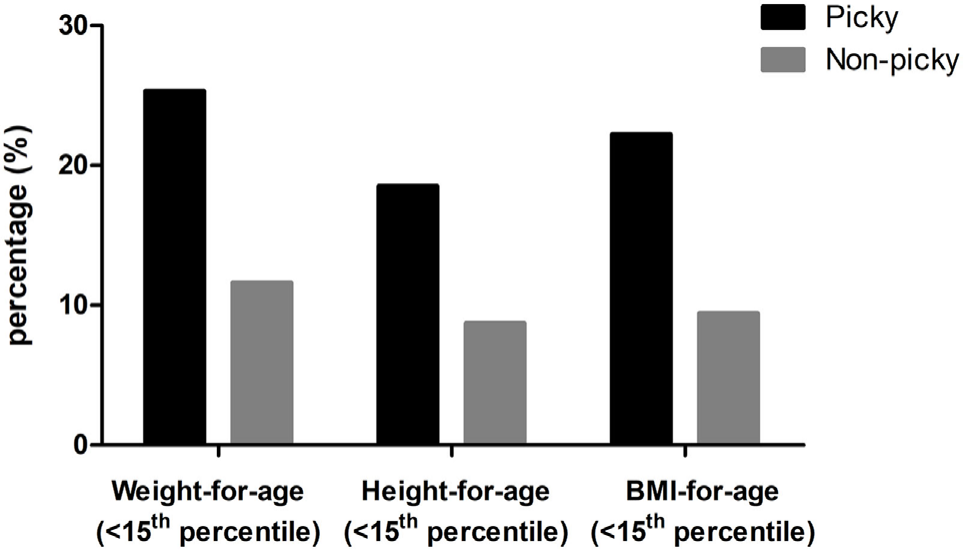
Percentages of children with weight for age, height for age, and body mass index (BMI) for age percentiles <10 in picky and non-picky groups.

The average weight, height, and BMI of this cohort of preschool children from Taiwan were 14.13 kg, 96.09 cm, and 15.30, respectively (Table 2). Each of these metrics was significantly lower in picky eaters than in non-picky eaters; picky eaters also had significantly lower average weight-for-age, height-for-age, and BMI-for-age percentiles. Moreover, mean weight- and height-for-age percentiles were both less than 50 (median of population) in picky eaters, whereas the weight-for-age, height-for-age, and BMI-for-age percentiles were all greater than 50 (median of population) in non-picky eaters. Compared to non-picky eaters, higher rates of weight-for-age, height-for-age, and BMI-for-age percentiles <15 was found in picky eaters (Figure 1). Significantly higher proportions of children with a weight-for-age percentile <15, a height-for-age percentile <15, and a BMI-for-age percentile <15 were picky eaters (*P* = 0.004, 0.023, and 0.005, respectively, Table 2). Average *z*-scores of weight-for-age, height-for-age, and BMI-for-age in picky eaters were all significantly lower than those *z*-scores of non-picky eaters (*P* < 0.001, Table 2).

**Table 2:** Growth and nutritional status in picky versus non-picky eaters.

Growth and nutritional status	Picky (*n* = 162)	Non-picky (*n* = 138)	95% CI^a^	*P*
			χ^2b^	
Weight, mean (SD)	13.13 (1.47)	15.30 (1.83)	−2.52, −1.82^a^	<0.001**
Weight-for-age percentile: mean (SD)	32.61 (14.04)	67.31 (15.43)	−39.79, −31.61^a^	<0.001**
<15th percentile: *n* (%)	41 (25.3)	16 (11.6)	8.238^b^	0.004*
Weight-for-age *z*-score	−0.46 ± 0.32	0.45 ± 0.42	−1.02, −0.82	<0.001**
Height, mean (SD)	93.77 (2.79)	98.81 (2.87)	−5.64, −4.45^a^	0.004*
Height-for-age percentile: mean (SD)	37.43 (14.23)	62.11 (15.07)	−27.76, −21.61	<0.001**
<15th percentile: *n* (%)	30 (18.5)	12 (8.7)	5.184^b^	0.023*
Height-for-age *z*-score	−0.41 ± 0.29	0.32 ± 0.41	−0.83, −0.64	<0.001
Body mass index (BMI), mean ± SD	14.93 (1.31)	15.67 (1.32)	−1.02, −0.46^a^	<0.001**
BMI-for-age percentile: mean (SD)	32.3 (14.47)	54.03 (14.91)	−24.84, −18.62^a^	<0.001**
<15th percentile: *n* (%)	36 (22.2)	13 (9.4)	8.025^b^	0.005*
BMI-for-age *z*-score	−0.33 ± 0.46	0.11 ± 0.40	−0.53, −0.35^a^	<0.001**

^a^Student’s t-test: *P < 0.05, **P < 0.001.

^b^Chi-square (χ^2^) analysis: *P < 0.05, **P < 0.001.

*CI, confidence interval*.

### Development

**Figure 2:**
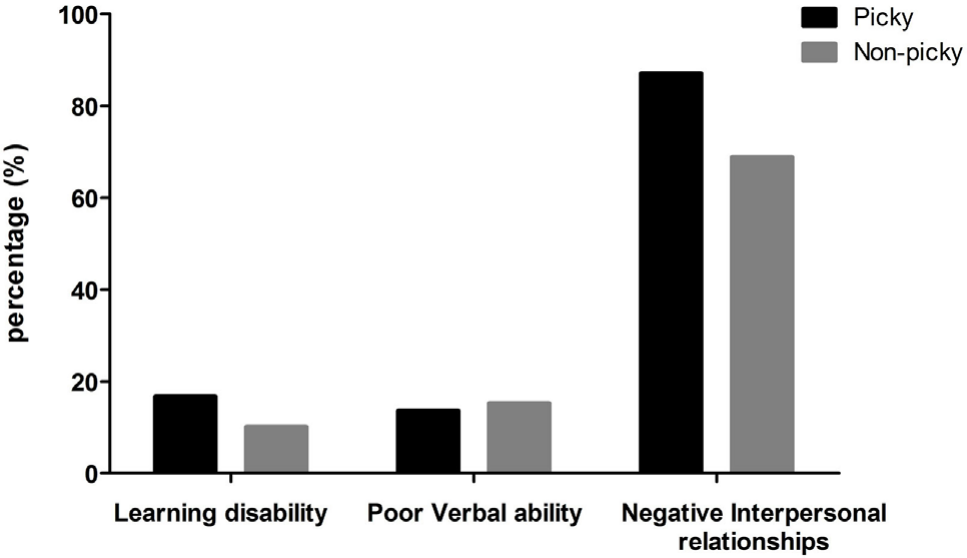
Prevalence of children with three categories of low quality development in picky and non-picky groups.

Table 3 displays differences in the development between picky eaters and non-picky eaters. Attention deficit and low learning ability were the detected low quality developments in learning ability. Slow verbal development and poor language development were the detected low quality developments in verbal ability. Afraid of unfamiliar places and afraid of being separated from relatives were the detected low quality developments in interpersonal relationships. Figure 2 demonstrates the prevalence rate of three categorized low quality developments in picky and non-picky group children. Of the three categories of low-quality development, a significantly higher prevalence of children who had negative interpersonal relationships was found in the picky group (*P* < 0.001, Table 3). A higher rate of “afraid of unfamiliar places” was reported to be picky eaters (*P* = 0.01, Table 3). Compared to non-picky group, a significantly lower score of all questionnaire items was found in the picky group (28.4 ± 4.1, vs. 22.8 ± 3.8, *P* < 0.001).

**Table 3:** Development in picky versus non-picky eaters.

Abnormal development:*n*(%)	Picky (*n* = 162)	Non-picky (*n* = 138)	χ^2a^	*P*
Learning disability	27 (16.7)	14 (10.1)	2.162	0.141
Attention deficit	17 (10.5)	8 (5.8)	1.581	0.209
Low learning ability	15 (9.3)	9 (6.5)	0.432	0.511
Poor verbal ability	22 (13.6)	21 (15.2)	0.057	0.817
Slow verbal development	15 (9.3)	16 (11.6)	0.223	0.637
Poor language development	14 (8.6)	12 (8.7)	0.036	0.850
Negative interpersonal relationships	141 (87)	95 (68.8)	13.638	<0.001**
Afraid of unfamiliar places	118 (72.8)	80 (58)	6.694	0.010*
Afraid of being separated from relatives	63 (38.9)	41 (29.7)	2.382	0.123

^a^Chi-square (χ^2^) analysis: *P < 0.05, **P < 0.001.

### Physical Activity and Health Status

Table 4 analyses differences in physical activity and health status between picky eaters and non-picky eaters. Of the categories of physical activity listed, a similar amount of low-level normal-pace walking was found, whereas low levels of other activities were proportionally higher among picky eaters, significantly so for stair-climbing (*P* = 0.038). Poor levels of physical activities were also significantly more common in picky eaters (*P* = 0.001), as were constipation (*P* = 0.044), and recent and/or more frequent acute infectious illnesses (*P* < 0.001). Compared to non-picky group, a significantly lower average score of the questionnaire of physical activities was found in the picky group (14.4 ± 2.3, vs. 12.3 ± 2.1, *P* < 0.001).

**Table 4:** Performance levels of physical activities and health status in picky versus non-picky eaters.

Variables:*n*(%)	Picky (*n* = 162)	Non-picky (*n* = 138)	χ^2a^	*P*
**Physical activities**				
(1) Normal-pace walking				
Low level	20 (12.3)	17 (12.3)	0.029	0.866
Normal	142 (87.7)	121 (87.7)		
(2) Sport activities				
Low level	31 (19.1)	16 (11.6)	2.663	0.103
Normal	131 (80.9)	122 (88.4)		
(3) Stair-climbing				
Low level	33 (20.4)	15 (10.9)	4.323	0.038*
Normal	129 (79.6)	123 (89.9)		
(4) Running				
Low level	22 (13.6)	13 (9.4)	0.880	0.348
Normal	140 (86.4)	125 (90.6)		

**General activities (1 + 2 + 3 + 4)**				
Poor (≥2 low-level activities)	50 (30.9)	20 (14.5)	10.269	0.001*
Good (≤1 low-level activity)	112 (69.1)	118 (85.5)		

**Medical illness**				
(1) Constipation	31 (19.1)	14 (10.1)	4.046	0.044*
(2) Acute infectious illness (3 months)				
>2 episodes	47 (29.1)	14 (10.1)	15.232	<0.001**
1 or 2 episodes	88 (54.2)	74 (53.6)	0.000	0.996
No episode	27 (16.6)	50 (36.3)	13.944	<0.001**

^a^Chi-square (χ^2^) analysis: *P < 0.05, **P < 0.001.

## Discussion

Although several studies have investigated the prevalence of picky eaters among children, few have assessed the association of picky eating with pediatric development, physical activity, and health status. This study provides an intriguing overview of picky eating in children aged 2–4 years in Taiwan and provides valuable insights concerning the impact on children’s growth, development, physical activity, and disease burden. The food preferences, development, and physical activities between picky eaters and non-picky eaters were scored and analyzed statistically. Our report is the first to correlate picky-eating behaviors with low-quality development and lower performance values of physical activities in preschool children.

Child’s picky eating was defined variably. There have been many studies of the prevalence of picky eating in childhood, and a large variation in prevalence was found (19). The majority of previous reports use “parental or caregiver’s conception of picky eater” to identify child’s picky eating. The picky eating defined in our study was based on the objective questionnaires of detecting picky eating by strong existence of picky eating behaviors on four questions. At 54%, the prevalence of picky eating in this study is relatively high compared to previous reports (6, 7, 20). A recent study indicated three significant parent-reported feeding questions that may identify persistent picky eaters at an early age (21). The three questions included a subjective identification of picky eater by parental conception, and two typical and common picky eating behaviors (strong likes with regard to food and unwillingness to accept new foods). These two picky eating behaviors were adopted in our questionnaire. Our study recruited the other two typical picky eating behaviors could help to identify picky eaters more precisely.

In a large survey of 7,057 children aged 2–7 years old in Hong Kong, 43% were reported by their parents as being picky eaters (20). A longitudinal study revealed that 40% of children’s picky-eating behavior lasted longer than 2 years (6). A cross-sectional survey found that the proportion of picky eaters increased from 19% at 4 months old to 50% by age 2 years (7). Another cross-sectional survey of Chinese preschoolers reported that prevalence of picky eating was higher in 24–35-month-olds (36%) compared to 6–11-month-olds (12%) (8). These findings suggest that picky eating is a chronic problem (6, 7). In our study, proportionally more participants in the younger age group were picky eaters (54.7% age 2–3 years vs. 53.2% age 3–4 years). Previous studies indicated that toddlerhood and preschool-age is the peak time for picky eating, and that pickiness seems to decline with age through early childhood (4, 22). The picky eaters in this cohort of preschool children from Taiwan observed significant lower numbers of accepted foods and lower values in the food preference. The items used in the questionnaire items for the assessment of picky eating in the present study was similar to those questionnaires of previous studies (7, 23, 24). In this study, picky eaters did not like to consume vegetables, meat, fruit, fish, and specific kinds of vegetables; similar picky-eating behaviors were observed among children in Hong Kong (20). The most common picky-eating behaviors in Taiwan were being unwilling to eat regular meals, refusing fruit and vegetables, and being likely to eat sweets or snacks instead of meals. These results are similar to picky-eating behaviors observed in another study in Singapore (23).

Excessive milk-drinking was a common picky-eating behavior found in our study. Based on the food records, excessive milk-drinking may induce a low appetite at meal-times and cause inadequate energy intake. A cross-sectional analysis in the United Kingdom, based on questionnaires completed by parents when their children were aged 30 months, indicated that eating a limited variety (17%) and preferring drinks to food (13%) were the most prevalent problem behaviors (3). The study found that an eating problem caused less weight gain over the first 2 years; 11.1% had weight loss compared with 3.5% of children who were not described as having an eating problem. The investigators concluded that weight loss is more common in picky eaters and excessive milk-drinking may be a cause of low appetite at meal-times.

Children with picky-eating habits have previously been identified as being at a potential risk for having nutritional deficits and the association of picky-eating behavior with growth status in children has also been evaluated (6, 15, 25). A longitudinal study of 120 children aged 2–11 years, detected no significant effects on growth (6). A cross-sectional survey in 1,498 children at 2.5, 3.5, and 4.5 years old, analyzed the relationship between eating behaviors, such as picky eating and dietary adequacy, and body weight; this study found that the amounts of energy, total fats, and protein consumed were significantly less for picky eaters than for non-picky eaters (energy, *P* = 0.0302, total fats, *P* = 0.0114; and proteins, *P* < 0.0001) (17). This study found that picky eaters were prone to consume fewer than two servings of meat and alternatives per day (odds ratio: 0.319; 95% CI 0.181–0.560). Further research found that picky eaters were twice as likely to be underweight at 4.5 years old as non-picky eaters (odds ratio: 2.415; 95% CI 1.383–4.216) (17).

There is still limited information about the impact of picky-eating behavior on the nutritional and growth status of preschool children. A study that compared the weight, height, and weight-for-height percentiles of 34 children with picky-eating behavior and 136 healthy controls found that seven of 34 children (20.6%) in the picky-eating group and nine of 136 (6.6%) in the control group were underweight (*P* = 0.02); being underweight was found in 15 children (14.2%) younger than 36 months old and in one child (1.6%) older than 36 months (*P* = 0.002) (26). The authors concluded that children with picky-eating habits are at an increased risk of being underweight, especially in those younger than 3 years old. Based on our data, weight and height of picky eaters were significantly lower than non-picky eaters: In general, the weight-for-age, height-for-age, and BMI-for-age percentiles of non-picky eaters were above 50th, while picky’ eaters were under 50th. Compared with non-picky eaters, *z*-score of weight-for-age, height-for-age, and BMI-for-age in picky eaters was 0.91, 0.73, and 0.44 SD lower, respectively (Table 2). Furthermore, picky-eaters comprised significantly higher proportions of children who were underweight, short, and with low BMI (<15 percentiles) compared to non-picky eaters.

A negative impact of picky eating behaviors on growth was found in pre-school and early school-age children. A Saudi study of 315 pre-school children with feeding problems comparing to 100 health control observed that the main feeding problems detected were picky eating in 85.5% of feeding problem with normal growth group, these group children were still having normal growth parameters, but they had significantly lower growth parameters than healthy children (12). In a China study of 937 recruited healthy children of 3–7 years old found the prevalence of picky eating as reported by parents was 54%; the weight for age *z*-score was significantly lower in picky eaters compared to non-picky eaters (8).

Picky eating behaviors may adversely affect dietary intake. A randomized trial of Chinese preschoolers (aged 2.5–5 years) using validated dietary analysis software of local database of recommended nutrient intakes found that median daily energy intake was 25% lower than the age-appropriate intake in preschool picky eaters, and indicated that preschool picky eaters with low weight-for-height index was at risk for significant dietary and nutrient insufficiencies (27). In that study, only near half of the picky eaters met the recommendation for daily servings of fruit, and fewer in vegetables (14.7%) and milk/milk products (6.3%), and grains and cereals (6.3%) (27). Another study in Chinese preschoolers (ages 3–7 years) found that picky eaters had lower intakes of protein, dietary fiber, vegetables, fish, and cereals, compared with non-picky eaters (8). A study of Chinese young infants and toddlers (6 months–3 years) observed that lower intake of eggs and their food group in picky eaters compared to non-picky eaters (9). In our study, the common dislike foods among preschool picky eaters (ages 2–4 years) were meat (37.1%), vegetables (38.9%), and fruit (22.2%).

A German study of 426 children aged 8–12 years found that picky eaters were more likely to exhibit problematic behaviors (such as withdrawal, somatic complaints, and anxiety/depression) than non-picky eaters (*P* = 0.001) (4). The relationship between several eating disorders (overeating, anorexia, or feeding difficulties) and development has been established in children and adolescents (28–33), whereas the relationship between picky-eating habits and development in preschool children has seldom been reported. Our study showed a positive correlation between picky-eating behaviors and low-quality general development. Furthermore, picky eaters tended to have a greater fear of unfamiliar places. The causal effect relationship between picky eating and low-quality of general development was unidentified due to lack of evidence in the research or literature. A future longitudinal study may lend a hand to illuminate the causal effect relationship between picky eating and low-quality of general development.

Research on the association between physical activity and dietary habits among adolescents indicates that physical activity levels are significantly positively correlated with fruit and vegetable intake (34). The association of picky-eating behaviors with physical activity and health status in preschool children has never been assessed. In our study, picky eaters tended to have lower values of the performance in physical activities especially a lower-level of stair-climbing. We also observed a positive correlation between picky-eating behaviors and risk of constipation and acute infectious illness in preschool children.

Oral nutritional supplementation promotes catch-up growth in picky eaters (35). Picky eaters (aged 3–5 years) with faltering growth who were randomized to receive 3-month oral nutritional supplementation had significantly greater increases in weight and height than non-supplemented controls, and developed proportionally fewer upper respiratory tract infections (35). Long-standing oral nutritional supplementation helped promote nutritional adequacy and growth in Filipino children who were at risk of nutritional deficiency (36). The results showed that long-term use of oral nutritional supplement improved food diversity and promoted adequate intake of nutrients that were inadequate in Filipino children’s diets without interfering the intake of normal family foods. A randomized controlled trial of Chinese preschoolers (ages 30–60 months) with picky eating behaviors and weight-for-height ≤25th percentile observed that changes in growth parameters and nutrient intake were significantly greater in the group with a nutritional milk supplement than the group with nutrition counseling alone (37). Compared to the children with nutrition counseling alone, increases in weight-for-age *z*-scores and weight-for-height *z*-scores were significantly greater at days 90 (all *P* < 0.05), and increases of intakes in energy, protein, carbohydrate, docosahexaenoic acid, arachidonic acid, calcium, phosphorous, iron, zinc, and vitamins (A, C, D, E, B6) were significantly higher at days 60 and 120 (all *P* < 0.01) in the children with a nutritional milk supplement (37).

Strength of this study included the population-based design in preschool children and extensive questionnaire to assess picky eating behavior, growth status, quality of development, level of physical activity, and health status. To minimize the selection bias, the participants enrolled were healthy, without economic burden for nutrition support; the caregivers had adequate concepts in children’s diets, development, and physical activities. To strengthen the scientific credibility, our study defined the picky eating by objective questionnaires and scored the performance on each questionnaire items of development and physical activity to demonstrate the difference of development and physical activities between picky and non-picky eaters.

Our study has possible limitations. The self-rating questionnaires only presented the point of views from caregivers, over-/underestimation in reporting may exist due to lack of objective behavioral observations on eating behaviors, interaction, developments, and physical activities. Finally, the inclusion of participants from three major cities may limit the generalization of the findings to the whole country.

## Conclusion

Picky-eating habits in preschool children may detriment development quality, physical activity level, and general health status. Parents need information and feeding strategies to increase the number of foods accepted by their toddlers and appropriate dietary interventions to develop sound feeding solutions to address picky-eating behaviors. Clinicians can help to guide parents on the best approaches to achieve better nutrition for children who are picky eaters.

## Availability of Data and Materials

Data for research purposes are available upon request.

## Consent for Publication

All participants gave consent for publication.

## Ethics Statement

Institutional Review Board of the Human Research Committee of Chang Gung Memorial Hospital approved the study protocol (Reference 97-0858B). All parents or guardians of children who were participants of the investigation provided their written informed consent.

## Author Contributions

HC contributed to the conception and design of the work. HC conducted the analysis and interpretation of the data. HC drafted the manuscript, reviewed the final manuscript, and did the final approval of the version to be published.

## Conflict of Interest Statement

The author declares that the research was conducted in the absence of any commercial or financial relationships that could be construed as a potential conflict of interest.

## Abbreviations

BMI, body mass index; CI, confidence interval.
